# Suicides in Mood Disorders in Psychiatric Settings in Nordic National Register–Based Studies

**DOI:** 10.3389/fpsyt.2020.00721

**Published:** 2020-07-23

**Authors:** Erkki T. Isometsä

**Affiliations:** Department of Psychiatry, University of Helsinki and Helsinki University Hospital, Helsinki, Finland

**Keywords:** suicide, mortality, incidence, depression, bipolar disorder, register, risk factor, systematic review

## Abstract

**Objective:**

Although risk factors for nonfatal suicidal behavior in mood disorders have been vastly investigated, rate and risk factors of suicide deaths are less well known. Extensive health care and other population registers in the Nordic countries (Denmark, Finland, Iceland, Norway, and Sweden) allow national-level studies of suicide rates and risk factors. This systematic review examined Nordic studies of suicide in mood disorders.

**Methods:**

National Nordic studies published after 1.1.2000 reporting on suicide mortality or relative risk in diagnosed unipolar depression or bipolar disorder treated in psychiatric settings; temporal variations in suicide risk after discharge, or risk factors for suicide were systematically reviewed.

**Results:**

Altogether 16 longitudinal studies reported on rate and risk of suicide in depression. They found 2%–8% of psychiatric inpatients with depression to have died by suicide. However, in Finland suicide risk among depressive inpatients halved since the early 1990s. Nine studies investigated suicide risk in bipolar disorder, finding 4–8% of patients having died by suicide in long term. The relative risk of suicide was consistently found extremely high (SMR > 100) during the first weeks postdischarge, declining steeply over time to approximately SMR of five after five years. Male gender, preceding suicide attempts, high severity of depression and substance abuse were found risk factors for suicide in depression, with only minor gender differences in risk factors, but major differences in lethal methods. Three studies investigated risk factors for suicide in bipolar disorder, finding male gender, preceding suicide attempts, and depressive episodes and psychiatric comorbidity to be associated with risk.

**Conclusions:**

Overall, of psychiatric inpatients with depressive of bipolar disorders in the Nordic countries, 2%–8% have died by suicide in the last few decades, but current rates may be lower. Suicide risk is approximately similar or somewhat higher among patients with bipolar disorder, risk factor studies of whom are fewer. Risk of suicide is remarkably high immediately after discharge, and higher among males than females, those with preceding suicide attempts, high severity of depression, or concurrent substance abuse. Generalizability of findings from these Nordic studies to other countries need to be investigated, and their methodological limitations understood.

## Introduction

Suicide is an important cause of death worldwide, and about 1.5% of all deaths are by suicide (1). About half of all suicides have been found to have suffered from mood disorders in psychological autopsy studies (2–5). Therefore, for suicide prevention overall, monitoring suicide mortality, knowledge of risk factors for suicide, and provision of adequate treatment for patients with mood disorders are central tasks.

Although risk factors for nonfatal suicidal behavior in mood disorders have been vastly investigated, rate and risk factors of suicide deaths are less well known (6). Suicides are rare events, and investigating their rate and risk factors necessitates massive patient samples. Over decades of work in this field, advances have been made in the knowledge of risk factors for suicide, and this information has been condensed into risk estimates in important meta-analyses (7, 8). However, as such documents reveal, for many important risk factors uncertainty prevails due to limitations of the samples available. Even though research on nonfatal suicidal acts can form an important proxy for studies of suicide deaths and complement view of significant risk factors, studies of suicide deaths are needed for confirming generalizability of findings between the populations (6). Early estimates for accumulating suicide mortality in major mood disorders were as high as 19% (9). However, already twenty years ago Bostwick and Pankratz (10) suggested an epidemiologically conceivable gradient of lifetime suicide risk (case fatality prevalence) from general population 0.5% to 8.6% in suicidal psychiatric inpatients. Nevertheless, scarcity of representative contemporary diagnosis-specific data is a major problem, despite a great need for monitoring rates of suicide in these central patient groups.

Due to availability of extensive health care and other population registers in the Nordic countries—Denmark, Finland, Iceland, Norway and Sweden—national-level studies of suicides in mood disorders are feasible (11), and can provide unbiased, generalizable information on suicide rates and risk factors. These countries share similarities in terms of population, culture, health care and social services, and health-related findings are in general comparable, with some but usually relatively small differences (12, 13). For purposes of this review, the most relevant difference has been higher male suicide mortality in Finland, which has markedly declined and approached, but not yet reached the general Nordic level (14).

The aim of this systematic review was to examine published literature on national-level or, in the absence of national-level data, large regional studies of suicide in psychiatric patients with unipolar and bipolar mood disorders from the Nordic countries from the year 2000 onwards.

## Methods

### Inclusion and Exclusion Criteria, Literature Searches and Collection of Relevant Papers

This review *included* nationally representative, register-based studies reporting on suicide mortality of diagnosed psychiatric patients with unipolar depression or bipolar disorder. Studies from the Nordic countries were included, if they were (a) based on national health care registers containing diagnostic data on patients’ with psychiatric hospitalizations and/or treatment as psychiatric outpatients; (b) concerned patients with ICD-10 codes F30-31 (manic episode or bipolar affective disorder) and F32-33 (depressive episode and recurrent depressive disorder), or similar diagnoses in the earlier versions of the ICD (ICD-9 or ICD-8); and (c) cause of death was reported based on linkage to nationally representative registers of official causes of death.

Studies were *excluded* if they were (a) not nationally representative, (b) did not contain diagnostically specific data on suicides in unipolar or bipolar mood disorders or the two groups combined, (c) classification of death as a suicide was not based on the official classification of causes of death in the country, or (d) contained exclusively primary health care patients. However, regionally representative data was considered, if nationally representative data was unavailable, and the study otherwise fulfilled the criteria above.

**Figure 1:**
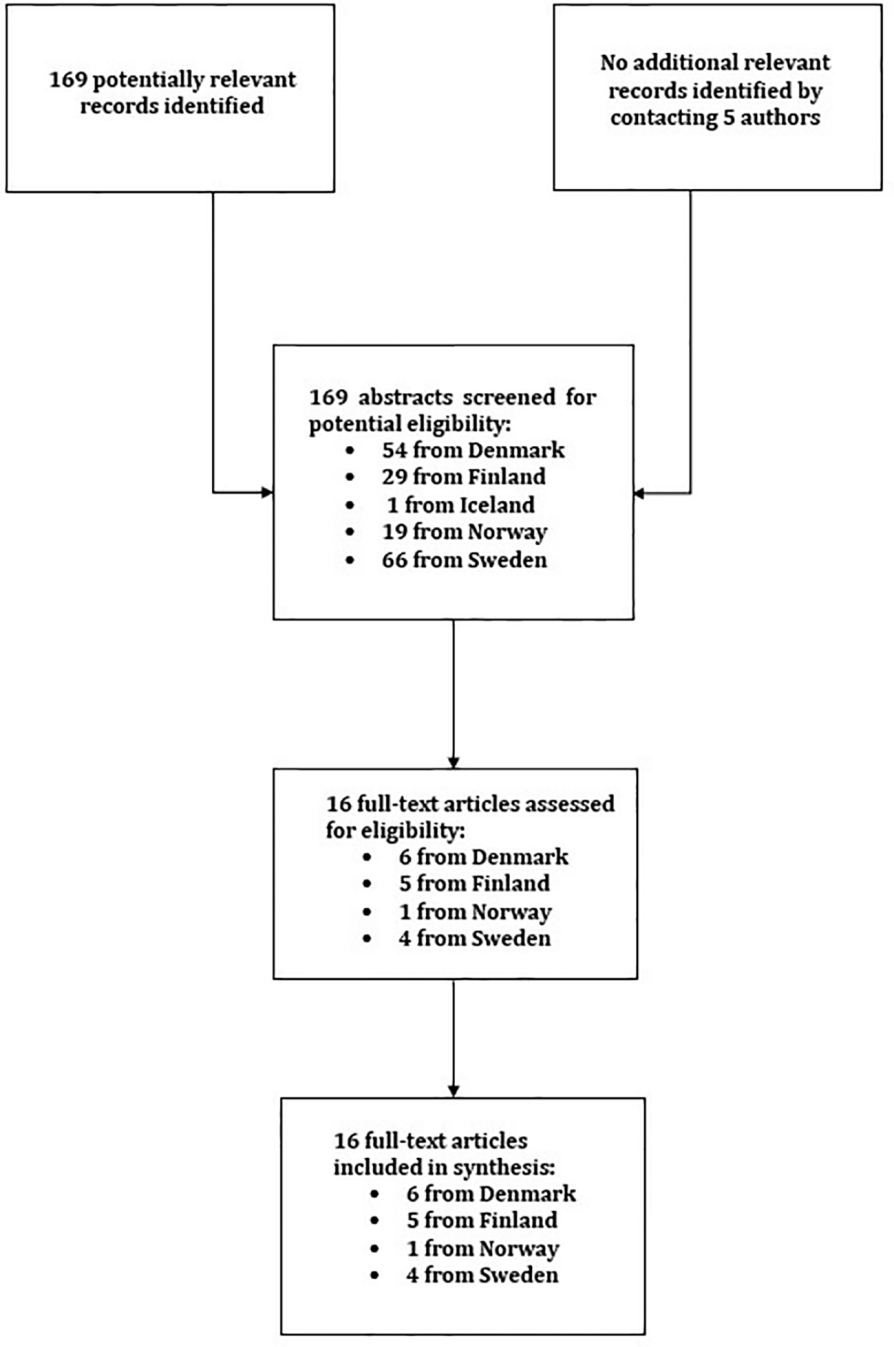
Flow chart of systematic literature search.

The author identified relevant studies from the PubMed published after 1.1.2000 by searching (on December 16^th^, 2019) using terms “suicide AND register* AND (depression OR bipolar disorder) AND [name of country]. These searches resulted in locating 169 potentially relevant abstracts (listed countrywise in the **Figure 1**). The author also contacted three Norwegian and two Icelandic authors to locate relevant papers, and sought references from other relevant papers. Of all these reports, total 16 were included as relevant for this review (see **Figure 1**). The remaining 153 were excluded as not nationally representative, or not containing diagnostically specific data on suicides in unipolar or bipolar mood disorders in psychiatric care. No primary health care studies emerged, so no studies were excluded as nonpsychiatric.

### Classification of Relevant Papers

The publications were divided into those reporting on (a) suicide mortality or relative risk in diagnosed unipolar depression treated in psychiatric settings; (b) such findings pertaining to diagnosed bipolar disorder; (c) temporal variations in suicide risk after discharge; (d) risk factors for suicide in unipolar depression or (e) bipolar disorder.

The author has organized the relevant 16 reports into **Tables 1**–**3**. As many studies reported on both unipolar and bipolar mood disorders, they are included in both contexts. The author extracted from the reports information case fatality prevalence, cumulative incidence, standardized mortality ratio (SMR) or other measures of absolute and relative mortality, plus information on several risk factors (gender, preceding suicide attempts, severity of illness at baseline, presence of psychotic features, comorbid substance abuse or dependence, and other). No statistical analyses were conducted.

**Table 1:** Suicide mortality in nationally representative register studies of unipolar depression in the Nordic countries.

Country and reference	Study Design	Ns of cohort and suicides with depression and registers used	Follow-up period	Suicide mortality/relative suicide risk (95% c.l.)^*^	Notes
**Denmark**					
Høyer et al. (15)	All first-time admissions for affective disorder in Denmark in 1973–1993	54,103 inpatients with affective disorder in the Danish Psychiatric Case Register, 3,127 suicides in National Register of Causes of Death	Up to end of 1993, maximum up to 21 years	SMR in unipolar depression 19.33 (18.37–20.06); in psychotic reactive depression 18.67 (17.47–19.95); in neurotic depression 10.51 (9.54–11.57). Risk highest during the first postdischarge year.	Relative risks presented for all diagnostic subgroups, but their specific numbers of patients and suicides not reported.
Qin and Nordentoft (16)	Nested case-control study of postdischarge suicides in Denmark in 1981–1997	423,128 population controls from the Integrated Database for Labour Market Research; 21,169 suicides in Cause of Death Register, 2,736 with affective disorders at hospitalization in the Danish Psychiatric Central Register	Up to 16 years	Sharply declining trends of adjusted risk ratio from first postdischarge week to >5 years: from 218.8 to 4.8 in men, and 1976.5 to 4.9 in women	Focus in postdischarge suicides, unipolar and bipolar disorders not differentiated
Nordentoft et al. (17)	Prospective study of incident suicides among Danes born 1955–1991 to end of 2006	176,347 mental health patients in the Danish Psychiatric Central Register, 46,233 with depression, 709 suicides in the Danish Registers of Causes of Death	Up to 36 years (median 18 years)	Cumulative incidence 6.67% (5.72–7.78) in men, 3.77% (3.05–4.66) in women	Out-patient visits included from 1995 to 2006
Leadholm et al. (18)	Prospective cohort of Danish in-patients with severe psychotic or nonpsychotic depression 1994–2010.	34,671 in- or out-patients in the Danish Central Psychiatric Register, 755 suicides in the Danish Cause of Death Register	Up to 17 years	2.1% nonpsychotic, 2.3% psychotic depression	Focus in severe depression and role of psychotic features for risk of suicide. Out-patients included from 1995 onwards.
Laursen et al. (19)	Prospective national Danish cohort 1995–2013	5,103,699 population; 554,987 with depression in the Danish Psychiatric Central Register and Danish National Patient Register; linked to Danish Registry of Causes of Death, N suicides not reported	Period 1995–2013	Mortality rate ratio (MRR) 4.66 (4.53–4.79) for suicides and accidents	Estimated reduced life expectancy 14.0 years in males and 10.1 years in females. Inpatients, plus out-patients from 1995 onwards.
**Finland**					
Suominen et al. (20)	Prospective cohort of Finnish inpatients hospitalized for depression and attempted suicide between 1996–2003	1,820 inpatients in the National Hospital Discharge Register, 106 suicides in cause of death register of the Statistics Finland	Up to 8 years (end of 2003, mean 4.2 years	6% died by suicide, crude incidence 1,390 per 100,000 patient-years	Cohort comprised only inpatients with depression, who had attempted suicide at index hospitalization; a high-risk population
Aaltonen et al. (21)	Prospective cohort of Finnish first-time inpatients with depression in 1991-2011; comparison of cohorts of 1991–1995 vs. 1996–2000 vs. 2001–2005 vs. 2006–2011	56,826 inpatients in the Finnish Hospital Discharge Register, 2,587 suicides in Statistic Finland’s register on causes of death.	Up to end of 2014, maximum 24 years (median 10.7 years)	Cumulative risk 6.13% (5.80–6.46); 8.4% (8.00–9.27) in men, 4.14% in women (3.83–4.45). Hazard ratios declined stepwise from 1.0 in 1991–1995 to 0.48 (0.43–0.55) in 2006-2011.	Reduction in risk despite halving of inpatient days in Finland between 1991 and 2011.
**Norway**					
Høye et al. (22)	Prospective cohort of inpatients with mood disorders in two northernmost counties in Norway in 1980–2012	Total 2,501 mood disorder patients in the University Hospital of North Norway and total 79 suicides in the Norwegian Cause of Death Registry; 1,656 with major depressive disorder and 47 suicides	Up to end of 2012 (total 13,766 person-years)	SMR for suicide 23.9 (18.0–31.8); in men 24.0 (17.0–33.9), in women 23.7 (14.3–39.4)	A high suicide mortality-area in Norway
**Sweden**					
Ösby et al. (23)	Prospective cohort of unipolar depressive inpatients in Sweden in 1973–1995	39,182 unipolar patients in the Swedish psychiatric inpatient register; 2,045 suicides in the cause of death register of Statistics Sweden.	Up to end of 1995, mean follow-up 9.37 years (SD ± 6.58) in men [1052 suicides/148372 person-years], 10.68 years (SD ± 6.65) in women [993 suicides/249366person-years]	SMR in men 20.9 (19.7-22.2), SMR in women 27.0 (25.3-28.7)	Suicides as part of overall mortality; SMRs rather than cumulative incidence
Tidemalm et al. (24)	Prospective cohort study of Swedes admitted to hospital in 1973–1982 for attempted suicide	39,695 people hospitalized for attempted suicide in the Swedish hospital discharge register, including 1,043 patients with bipolar or unipolar disorder with 271 suicides during follow-up in the cause of death register.	Follow-up to 2003 (for 21–31 years)	Adjusted hazard ratio for suicide in men 3.5 (3.0-4.2), in women 2.5. (2.1-3.0)	Cohort comprised only patients having attempted suicide. Two-thirds (68%) of the hospitalized patients were not given a psychiatric diagnosis and comprised the reference group of the study.
Haglund et al. (25)	Prospective cohort of all discharges of inpatients in Sweden in 1973–2009	Overall 2,883,088 discharges in the Swedish National Patient Register and 3,695 suicides in the Cause of Death Register; 447,254 and 1,177 with depression, respectively	First 30 days after discharge	Suicide rate 3,600 (3390-3800) per 100,000 patient-years in depression	Focus in immediate first-month postdischarge suicides, the period with highest risk

*Incidence rates converted to per 100,000 patient years for comparability. SMR, standardized mortality ratio.

**Table 2:** Suicide mortality in bipolar disorder in Nordic national-level register-based studies.

Country and reference	Study Design	Ns of cohort and suicides with bipolar disorder	Follow-up period	Suicide mortality/relative suicide risk (95% c.l.)^*^	Notes
**Denmark**					
Høyer et al. (15)	All first-time admissions for affective disorder in Denmark in 1973–1993	54,103 inpatients with affective disorder in the Danish Psychiatric Case Register, 3,127 suicides in National Register of Causes of Death	Up to end of 1993, maximum up to 21 years	SMR in bipolar disorder 18.09 (16.32–20.07). Risk highest during the first postdischarge year.	Relative risks presented for all diagnostic subgroups, but their specific numbers of patients and suicides not reported.
Qin and Nordentoft (16)	Nested case-control study of postdischarge suicides in Denmark in 1981–1997	423,128 population controls the Integrated Database for Labour Market Research; 21,169 suicides in Cause of Death Register, 2,736 with affective disorders at hospitalization in the Danish Psychiatric Central Register.	Up to 16 years	Sharply declining trends of adjusted risk ratio from first postdischarge week to >5 years: from 218.8 to 4.8 in men, and 1976.5 to 4.9 in women	Focus in postdischarge suicides, unipolar and bipolar disorders not differentiated
Nordentoft et al. (17)	Prospective study of incident suicides among Danes born 1955–1991 to end of 2006	176,347 mental health patients in the Danish Psychiatric Central Register, 5,927 with bipolar disorder, 175 suicides in the Danish Registers of Causes of Death.	Up to 36 years (median 18 years)	Cumulative incidence 7.77% (6.01–10.05) in men, 4.78% (3.48–6.56) in women	Out-patient visits included from 1995 to 2006
**Finland**					
Isometsä et al. (26)	Prospective cohort of all hospitalizations for bipolar disorder in Finland in 1987–2003.	Total 52,747 hospitalizations in the Finnish Hospital Discharge Register, 466 suicides in the cause of death register of Statistics Finland.	One year after discharge (end of 2003)	Incidence 492 (449–539)/100,000 patient-years; 694 (618–778) in males and 332 (286–385) in females	Unit of analysis hospitalization, focus on first year after hospitalization; a high-risk period
Toffol et al. (27)	Prospective cohort of inpatients hospitalized for bipolar disorder and attempted suicide between 1996 and 2003	826 inpatients in the Hospital Discharge Register, 50 suicides in the causes of death register of the Statistics Finland	Up to 8 years (end of 2003), mean 3.5 years	6.1% died by suicide, crude incidence 1,700 per 100,000 patient-years	Cohort comprised only inpatients with bipolar disorder, who had attempted suicide at index hospitalization; a high-risk population.
**Norway**					
Høye et al. (22)	Prospective cohort of inpatients with mood disorders in two northernmost counties in Norway in 1980–2012	Total 2,501 mood disorder patients in the University Hospital of North Norway and total 79 suicides in the Norwegian Cause of Death Registry; 845 with bipolar disorder and 32 suicides	Up to end of 2012 (total 10,272 person-years)	SMR for suicide 22.8 (16.1–32.2); in men 16.1 (9.4–27.8), in women 31.8 (20.3–49.8)	A high suicide mortality-area in Norway
**Sweden**					
Ösby et al. (23)	Prospective cohort of bipolar inpatients in Sweden in 1973–1995	15,386 bipolar inpatients in the Swedish psychiatric inpatient register, 672 suicides in the cause of death register of Statistics Sweden.	Up to end of 1995, mean follow-up 10.52 years (SD ± 6.69) in men [345 suicides/69,222 person-years], 11.51 years (SD ± 6.73) in women [327 suicides/101,393 person-years]	SMR in men 15.0 (13.5–16.7), SMR in women 22.4 (20.0–24.9)	Suicides as part of overall mortality; SMRs rather than cumulative incidence
Hansson et al. (28)	Prospective cohort study based on the Swedish National Quality Register for Bipolar Affective Disorder (BipoläR) and suicide deaths in 2004–2014.	12,850 bipolar psychiatric out-patients in the BipoläR, 90 suicides in Cause of Death Register	Up to end of 2014, median follow-up 3.80 years	55 suicides (1.14%) among 4,844 male patients; 35 suicides (0.44%) among 8,006 female patients	Study based on a national quality register, from which they can opt out.
Haglund et al. (25)	Prospective cohort of all discharges of inpatients in Sweden in 1973–2009	Overall 2,883,088 discharges in the Swedish National Patient Register and 3,695 suicides in the Cause of Death Register; 119,948 and 147 with bipolar disorder, respectively	First 30 days after discharge	Suicide rate 1,740 (1,470–2,040) per 100,000 patient-years in bipolar disorder	Focus in immediate first-month postdischarge suicides, the period with highest risk

^*^Incidence rates converted to per 100,000 patient years for comparability. SMR, standardized mortality ratio.

**Table 3:** Risk factors for suicide in mood disorders in Nordic national-level register-based studies.

Unipolar depression									
			Risk factor and adjusted relative risk (with 95% confidence intervals)						
	Study design	Ns of patients and suicides	Male gender	Preceding suicide attempts	Severity of depression at baseline	Psychotic features	Comorbid substance abuse	Other	Notes
Study									
Høyer et al. (29)	Postdischarge suicides (< 1y) of patients with affective disorder in Denmark in 1994–1995, matched controls	135 suicide cases, 135 controls identified through the Danish Psychiatric Central Register.	(matched for gender)	IRR 4.97 (2.40–10.27)	–	–	–	Loss of job in < 12 months, IRR 2.94 (1.16–7.46); Clinical improvement during admission 0.33 (0.15–0.74); Antidepressant 0.35 (0.16–0.75)	Included 30 suicides and 30 controls with bipolar disorder
Suominen et al. (20)	Prospective cohort of Finnish inpatients hospitalized for depression and attempted suicide between 1996–2003	1,820 inpatients in the National Hospital Discharge Register, 106 suicides in cause of death register of the Statistics Finland	HR 2.04 (1.39–3.03)	–	Moderate HR 1.17 (0.26–5.29); Severe 3.10 (0.75–12.81)	HR 3.32 (1.95–5.67)	–	Antidepressants HR 1.06 (0.71–1.58)	Cohort comprised only inpatients with depression, who had attempted suicide at index hospitalization; a high-risk population
Nordentoft et al. (17)	Prospective study of incident suicides among Danes born 1955–1991 to end of 2006	46,233 mental health patients with depression in the Danish Psychiatric Central Register, 709 suicides in the Danish Registers of Causes of Death	Cumulative incidence 6.67% (5.72–7.78) in men, vs. 3.77% (3.05–4.66) in women	Cumulative incidence 10.48% (8.24–13.32) in men, 6.51% (5.23–8.09) in women	–	–	Cumulative incidence 6.74% (5.24–8.67) in men, 7.12% (4.68–10.83) in women	–	Out-patient visits included from 1995 to 2006
Leadholm et al. (18)	Prospective cohort of Danish in-patients with severe psychotic (PD) or nonpsychotic (non-PD) depression	34,671 in- or out-patients in the Danish Central Psychiatric Register, 755 suicides in the Danish Cause of Death Register	non-PD AOR 1.89, PD AOR 1.98	non-PD AOR 5.02, PD AOR 5.17	–	2.1% nonpsychotic vs. 2.3% psychotic depression; AOR 0.97 (0.83–1.15)	–	Age at diagnosis PD AOR 1.1 (1.0–1.1); non-PD 1.0 (1.0–1.1)	Focus in severe depression and role of psychotic features for risk of suicide
Aaltonen et al. (30)	Prospective cohort of first-time inpatients with depression in 1991–2011 in Finland	56,826 inpatients in the Finnish Hospital Discharge Register, linked with Census Register of Statistics Finland, and with 2,587 suicides in Statistic Finland’s register on causes of death.	AHR 2.07 (1.91–2.24)	At admission AHR 2.110 (1.862–2.391); previous 4 years AHR 2.111(1.845–2.415)	Severe vs. moderate AHR 1.188 (1.083–1.303)	Psychotic vs. moderate AHR 1.451 (1.301–1.619)	Alcohol dependence AHR 1.261 (1.129–1.409)	Tertiary vs basic education AHR 1.295 (1.160–1.445); Highest vs. lowest income AHR 1.191 (1.068–1.329); Living alone AHR 1.124 (1.029–1.227)	Follow-up to maximum 24 years. Suicide mortality halved between 1991 and 1995 vs. 2006 and 2011
Study									
Nordentoft et al. (17)	Prospective study of incident suicides among Danes born 1955–1991 to end of 2006	5,927 mental health patients with bipolar disorder in the Danish Psychiatric Central Register, 175 suicides in the Danish Registers of Causes of Death	Cumulative incidence 7.77% (6.01-10.05) in men, vs. 4.78% (3.48–6.56) in women	Cumulative incidence 17.08% (11.19–26.07) in men, vs. 9.39% (6.07–14.54) in women			Cumulative incidence 10.01% (6.40–15.66) in men, vs. 5.20% (2.81–9.60) in women		Out-patient visits included from 1995 to 2006
Isometsä et al. (26)	Prospective cohort of all hospitalizations for bipolar disorder in Finland in 1995-2003.	35,946 hospitalizations in the Finnish Hospital Discharge Register, 129 suicides within 120 days from discharge in the causes of death register of the Statistics Finland; 65 after hospitalization for depressive, 28 after mania, 20 after mixed episodes	After depressive HR 3.63 (2.12-6.23)	After depressive HR 8.05 (2.49–26.04) for attempts at index hospitalization	Depressive > mixed > manic > other index hospitalization			After depressive hospitalization HR for lithium 0.186 (0.07–0.52)	
Hansson et al. (28)	Prospective cohort study based on the Swedish National Quality Register for Bipolar Affective Disorder (BipoläR) and suicide deaths in 2004-2014.	12,850 bipolar out-patients in the BipoläR, 90 suicides in Cause of Death Register	Hazard ratio 2.56 (1.68–3.92)	HR 4.10 (2.43–6.92)	Any affective episode in the previous year HR 2.39 (1.47–3.88); any depressive episode in the previous year HR 2.24 (1.25–4.01)		Comorbid substance use disorder HR 3.79 (2.21–6.50)	Living alone HR 2.45 (1.36–4.43); criminal conviction HR 4.43 (1.35–14.53); any comorbid psychiatric disorder HR 2.64 (1.69–4.13); comorbid anxiety disorder HR 1.91 (1.07–3.42); psychiatric inpatient care HR 2.79 (1.62–4.80); involuntary commitment HR 3.50 (1.71–7.15).	Study based on a national quality register, from which they can opt out.

IRR, incidence rate ratio; HR, hazard ratio; AHR, adjusted hazard ratio; OR, odds ratio; AOR, adjusted odds ratio.

## Results

### Suicide Deaths in Unipolar Depression

Altogether eleven Danish (15–19), Finnish (20, 21), Norwegian (22), and Swedish (23–25) studies reported on rate and risk of suicide in unipolar depression (**Table 1**). They found between 2% and 8% of psychiatric inpatients with depression to have died by suicide. The Danish study by Nordentoft et al. (17) was the most long-term (median follow-up 18 years), finding about 7% of males and 4% to have died by suicide. However, a Finnish national study (21) demonstrated decline of hazard ratio for suicide to 0.48 among depressive inpatients since the early 1990s (**Table 1**).

### Suicide Deaths in Bipolar Disorder

Eight studies reported on suicide risk in bipolar disorder (**Table 2**). Three of them were Danish (15–17), two Finnish (26, 27) and one from Norway (22) and three from Sweden (23, 25, 28). In general, these studies found 4% to 8% of their bipolar patients having died by suicide. The study of Hansson et al. (28) was included, even though it is based on a national Swedish quality register (BipoläR), from which patients can (by law) opt out. This study found significantly lower suicide mortality in 2004–2014 among patients included (**Table 2**).

### Temporal Variations in Suicide Risk After Discharge

For both unipolar and bipolar patients, the relative risk of suicide was consistently found extremely high (SMR > 100) during the first weeks postdischarge (**Tables 1** and **2**), declining then over time to approximately SMR of five after five years the last hospitalization (16, 25, 26).

### Risk Factors for Suicide in Unipolar Depression

The five studies reporting on risk factors for suicide (17, 18, 20, 29, 30) consistently found male gender, preceding suicide attempts, high severity of depression and substance abuse risk factors for depression in long term. Aaltonen et al. (30) in Finland investigated also gender differences in thirteen risk factors, finding only small differences in them, but major differences in lethal methods used. A Danish study (18) focused on psychotic vs. severe nonpsychotic depressions, finding no significant difference in risk (**Table 3**).

### Risk Factors for Suicide in Bipolar Disorder

Risk factors for suicide in bipolar disorder were reported by three studies (17, 26, 28), which found male gender, preceding suicide attempts, and hospitalization for depressive and mixed episodes to associated with risk (**Table 3**). The study of Hansson et al. (28) was included, even though it is based on a national Swedish quality register (BipoläR), from which patients can opt out. In this study the researchers were able to investigate role of 20 putative risk factors for suicide, documenting role of male gender, living alone, criminal conviction, previous year depressive episodes, psychiatric comorbidity (substance abuse, anxiety or personality disorders) and severity indicators of psychiatric history (inpatient treatment, involuntary commitment) as risk factors (**Table 3**).

## Discussion

### Main Findings

Overall, findings of the available national diagnosis-specific studies from Denmark, Finland and Sweden were broadly consistent. Of psychiatric inpatients with depressive of bipolar disorders in the last few decades, 2%–8% have died by suicide in long term. However, these estimates generally concern only psychiatric patients ill enough to be hospitalized. Furthermore, suicide mortality has markedly declined among depressive inpatients in Finland after the early 1990s. Given decline in overall suicide mortality in all of the Nordic countries, suicide mortality in mood disorder patients has likely declined in all of them. Suicide risk is approximately similar or somewhat higher among patients with bipolar disorder, of whom differences in mortality between type I or II patients remain obscure.

### Temporal Variation in Risk

Rate of suicides is very strongly related to phase of illness and treatment. Incidence of suicide in the first weeks after discharge from a psychiatric hospital has been consistently found to be very high, even over hundred times that of the general population. In the Swedish national study from 1973 through 2009 by Haglund et al. (25) suicide rate among depressive inpatients in the first month after discharge was 3,600 and among bipolar inpatients 1,740 per 100,000 patient-years. Findings from Denmark (16) and Finland (26) are broadly similar, and in accordance with the broader international literature (31, 32). Although the postdischarge treatment phase is a well-known period of high risk of suicide across diagnostic groups, the documented role of illness factors need to be noted. Haglund et al. (25) found marked differences in rates between diagnostic groups, and Isometsä et al. (26) found marked differences in temporal patterns of risk between hospitalizations for different types of index episodes among bipolar patients. After the immediate postdischarge weeks, suicide risk generally steeply declines over time, typically reaching level of about fivefold suicide mortality when over five years has passed after the last hospitalization (31). For suicide prevention, this translates into a temporal window of very high risk to which preventive efforts (improving continuity of treatments between settings, monitoring of suicide risk and safety planning), need to be focused. However, it is somewhat surprising, that despite this well-known high-risk period and availability of large-scale data, short-term risk factors during this period have remained largely uncharted. Almost all studies focus on risk factors during long-term follow-up. They may or may not be similar.

### Risk Factors and the Role of Gender

In these Nordic studies of depression, long-term risk of suicide was found markedly higher among males than females, those with preceding suicide attempts, high severity of depression at outset of illness, or concurrent substance abuse at baseline. The finding of males with depression having higher risk for suicide than females is fully consistent with the broader suicide literature. The Finnish national study by Aaltonen et al. (30) is the largest of the depression studies, and was large enough to compare potency and prevalence of thirteen risk factors between genders. The observed gender differences were found unremarkable, whereas differences lethal methods pronounced, making them the likely explanation for the higher male suicide mortality in mood disorders. Although rate of suicide in bipolar disorder was recorded in multiple studies, those or risk factors for suicide in it were scarce. The three studies (17, 26, 28) which investigated risk factors for suicide in bipolar disorder generally found male gender, preceding suicide attempts, and occurrence or hospitalization for depressive episodes to be associated with risk. The quality register-based Swedish study (28) found no difference in risk between types of bipolar disorder, but documented risk related to any types of comorbid disorders, living alone and criminal convictions.

### Previous Attempts as Indicators of High Risk

A nonfatal suicide attempt is the strongest known indicator of suicide risk. Therefore, it is unsurprising, that a preceding suicide attempt emerged as a very strong predictor of suicide among patients with mood disorders. The trajectories of higher risk seemed to persist over time. The clinical importance of this highly consistent finding is obvious, as the cumulative incidence of suicides, particularly among males, approached 20% at worst (17, 30). However, to some extent these findings may be inflated by methodological factors, as information on all lifetime suicide attempts is usually unavailable, and only on those severe enough to result into hospital treatment, and temporally associated with the index hospitalization or the few preceding years is known. Methodological differences between studies in these factors are also likely to explain differences in observed findings. Since use of violent methods may involve much higher risk of future suicide death than overdoses (33–35), future studies should also examine risk based on the method used in the preceding nonfatal attempts.

### Role of Illness Severity

Risk of suicide in mood disorders is related to severity of illness. Patients with severe or psychotic illness had higher risk of suicide, and this higher risk persists for years or even decades. It is important to note, that this is true even though information was almost always available only for severity of the first index or baseline episode. Severity of subsequent episodes, not to speak of state of illness at the time of death remained obscure. However, psychological autopsy studies of patients with depression generally find suicides to occur at a time when a depressive episode is ongoing (36), and clinical studies of suicide attempts among depressive patients find suicidal acts to cluster in illness episodes (37–39). It is therefore credible although uncertain, that suicides among depressive patients occur during depressive episodes. Higher severity or psychotic features at index episodes may predict not only similarity of future episodes (40), but also higher rate of recurrences and chronicity of illness, i.e. more time at high-risk clinical states over time (41). However, although patients with psychotic features have highest risk for suicide among those with depression, this is probably due to the higher severity of depressive symptoms overall, rather than psychotic features per se (18, 20, 30). In these register-based studies, there are only small differences in observed risk between severe nonpsychotic and psychotic depression. Whether more accurate and episode-specific analyses will demonstrate differences in risk remains to be solved by future studies.

### Limitations of Register-Based Studies

Although these findings are based on population studies rather than samples and likely very generalizable beyond the Nordic countries to other countries and settings, it is also important to understand the context and important limitations of register–based studies in study of suicide, in particular crudeness of the data available. Most (75%) of the studies reviewed comprises exclusively in-patient populations, which are known to represent tip of the iceberg in clinical epidemiology (42). Therefore, rates of suicide are likely to be overestimate rates compared with outpatient settings. All the measures in a national register are based on routine clinical evaluations, accuracy of which may be variable. In most cases, this likely results in random measurement errors, which will deflate subgroup differences. Furthermore, in most of the longitudinal studies data on individuals’ characteristics is collected from the life situation at the time of study baseline, whereas situation at the time of suicide at the time of death remains obscure. The paradoxical findings of higher income, education or being married and employed as predictors for suicide among psychiatric patients with mood disorders in the Nordic settings are likely explained by later losses, which remain unknown. The Danish study of individual trajectories by Agerbo (43) clarified such paradoxical findings by demonstrating, that it is precisely loss of these characteristics over time, which is related to suicide risk. Generalizability of these findings to other countries need to be investigated, as e.g. employment, education and income may be more important determinants of access to treatment outside the Nordic countries. Overall, generalizability of all the findings of these Nordic studies to other countries needs to be carefully examined.

The crudeness of register data is revealed by comparing it with comprehensive clinical-epidemiological studies. For example, the reported rates of psychiatric comorbidity are minimal in register data compared with such clinical cohorts (44, 45). Since information on longitudinal course of illness is usually totally missing, the remarkably strong association of clinical state, i.e. presence or absence of illness episodes with suicidal acts (37–39) remains unobserved. Furthermore, with only a few exceptions (30), register-based studies cover only a few of the tens of putative risk factors in depression (6), and large-scale studies informing risk factors related to suicide deaths in bipolar disorder are scarce (7), particularly pertaining type II. A noteworthy exception and perhaps a harbinger of future epidemiology is the relative richness of data in the Swedish bipolar quality register BipoläR (28). Overall, currently available register-based studies are important in providing epidemiologically credible estimates of total suicide risk over time among those most ill, and generalizable information on some easily-observed and temporally static risk factors, but not a complete picture of the numerous factors influencing suicide risk.

### Past Decades vs. Current Treatment Settings

It is important also to note the differences in time periods covered between the studies. Several of the included studies covered time from 1970s onwards, as from that time register data is available. However, studies from the 1970s and 1980s cover a quite different era of treatments and service provision. In 11 of the 16 studies included into the Tables the follow-up reached the current millennium, and only five (19, 21, 22, 28, 30) the 2010s, latest up to 2014 (28). It is unavoidable, that long-term follow-up studies cover past decades, but there is also a significant delay in updating register-based datasets. As Aaltonen et al. (21) verified, besides the general decline in suicide rates in the Nordic countries, specifically also the suicide mortality of in patients with depression has halved at least in Finland from the 1990s to the 2010s. Thus, the suicide mortality estimates reported here most likely exceed current realities, and the degree to which the architecture of risk factors has changed over time along with societal changes and changes in treatment and their provision remains an important future research topic. Obviously, there is a great need to update the current knowledge base.

### Future Prospects: Electronic Health Records, Quality Registers, and Big Data

Health care records are undergoing transformation, and electronic records and quality registers (see ref. (28) may in the future contain an order of magnitude richer source of data for monitoring treatment quality, outcome and suicide research. In particular, availability of repeated symptom scores plus trait measures concurrently with updated information on family situation and employment will result in more accurate view of factors influencing suicide risk (46–48). More sophisticated machine learning tools may be helpful in uncovering previously unobserved patterns related to risk, but statistical rarity of suicide will unavoidably limit predictive ability also in the future, irrespective of sophistication of analytical tools (49). Coverage plus validity and uniformity of measures across settings will be central issues for utility of data. Availability of such data will hopefully advance efforts to prevent suicides among patients suffering from mood disorders.

## Data Availability Statement

The original contributions presented in the study are included in the article/supplementary materials; further inquiries can be directed to the corresponding author.

## Author Contributions

The author confirms being the sole contributor of this work and has approved it for publication.

## Conflict of Interest

The author declares that the work was conducted in the absence of any commercial or financial relationships that could be construed as a potential conflict of interest.

## References

[1] Naghavi M Global Burden of Disease Self-Harm Collaborators Global, regional, and national burden of suicide mortality 1990 to 2016: systematic analysis for the Global Burden of Disease Study 2016. BMJ (2019) 364:l94. 10.1136/bmj.l94 31339847 PMC6598639

[2] Cavanagh JT Carson AJ Sharpe M Lawrie SM Psychological autopsy studies of suicide: a systematic review. Psychol Med (2003) 33(3):395–405. 10.1017/S0033291702006943 12701661

[3] Arsenault-Lapierre G Kim C Turecki G Psychiatric diagnoses in 3275 suicides: a meta-analysis. BMC Psychiatry (2004) 4:37. 10.1186/1471-244X-4-37 15527502 PMC534107

[4] Conner KR Bridge JA Davidson DJ Pilcher C Brent DA Metaanalysis of Mood and Substance Use Disorders in Proximal Risk for Suicide Deaths. Suicide Life Threat Behav (2019) 49(1):278–92. 10.1111/sltb.12422 PMC8378507 29193261

[5] Cho SE Na KS Cho SJ Im JS Kang SG Geographical and temporal variations in the prevalence of mental disorders in suicide: Systematic review and meta-analysis. J Affect Disord (2016) 190:704–13. 10.1016/j.jad.2015.11.008 26600412

[6] Isometsä E Suicidal behavior in mood disorders – who, when and why? Can J Psychiatry (2014) 59:120–30. 10.1177/070674371405900303 PMC4079239 24881160

[7] Hawton K Casañas I Comabella C Haw C Saunders K Risk factors for suicide in individuals with depression: a systematic review. J Affect Disord (2013) 147(1-3):17–28. 10.1016/j.jad.2013.01.004 23411024

[8] Schaffer A Isometsä ET Tondo L Moreno D Turecki G Reis C International Society for Bipolar Disorders Task Force on Suicide: Meta-analyses and Meta-Regression of Correlates of Suicide Attempts and Suicide Deaths in Bipolar Disorder. Bipolar Disord (2015) 17:1–16. 10.1111/bdi.12271 PMC6296224 25329791

[9] Goodwin FK Jamison KR Manic-Depressive Illness. New York: Oxford University Press (1990).

[10] Bostwick JM Pankratz VS Affective disorders and suicide risk: a reexamination. Am J Psychiatry (2000) 157:1925–32. 10.1176/appi.ajp.157.12.1925 11097952

[11] Erlangsen A Qin P Mittendorfer-Rutz E Studies of Suicidal Behavior Using National Registers. Crisis (2018) 39(3):153–8. 10.1027/0227-5910/a000552 29792362

[12] Nordic Burden of Disease Collaborators Life expectancy and disease burden in the Nordic countries: results from the Global Burden of Diseases, Injuries, and Risk Factors Study 2017. Lancet Public Health (2019) 4(12):e658–69. 10.1016/S2468-2667(19)30224-5 PMC7098475 31759894

[13] Nordentoft M Wahlbeck K Hällgren J Westman J Ösby U Alinaghizadeh H Gissler M Excess mortality, causes of death and life expectancy in 270,770 patients with recent onset of mental disorders in Denmark, Finland and Sweden. PLoS One (2013) 8:e55176. 10.1371/journal.pone.0055176 23372832 PMC3555866

[14] Nordic Medico-Statistical Committee Health Statistics for the Nordic Countries 2017. Copenhagen: Nomesco p. 108, (2017).

[15] Høyer EH Mortensen PB Olesen AV Mortality and causes of death in a total national sample of patients with affective disorders admitted for the first time between 1973 and 1993. Br J Psychiatry (2000) 176:76–82. 10.1192/bjp.176.1.76 10789332

[16] Qin P Nordentoft M Suicide risk in relation to psychiatric hospitalization: evidence based on longitudinal registers. Arch Gen Psychiatry (2005) 62(4):427–32. 10.1001/archpsyc.62.4.427 15809410

[17] Nordentoft M Mortensen PB Pedersen CB Absolute risk of suicide after first hospital contact in mental disorder. Arch Gen Psychiatry (2011) 68(10):1058–64. 10.1001/archgenpsychiatry.2011.113 21969462

[18] Leadholm AK Rothschild AJ Nielsen J Bech P Ostergaard SD Risk factors for suicide among 34,671 patients with psychotic and non-psychotic severe depression. J Affect Disord (2014) 156:119–25. 10.1016/j.jad.2013.12.003 24388683

[19] Laursen TM Musliner KL Benros ME Vestergaard M Munk-Olsen T Mortality and life expectancy in persons with severe unipolar depression. J Affect Disord (2016) 193:203–7. 10.1016/j.jad.2015.12.067 26773921

[20] Suominen K Haukka J Valtonen HM Lönnqvist J Outcome of patients with major depressive disorder after serious suicide attempt. J Clin Psychiatry (2009) 70(10):1372–8. 10.4088/JCP.09m05110blu 19906342

[21] Aaltonen KI Isometsä E Sund R Pirkola S Decline in suicide mortality after psychiatric hospitalization for depression in Finland between 1991 and 2014. World Psychiatry (2018) 17(1):110–2. 10.1002/wps.20501 PMC5775129 29352527

[22] Høye A Nesvåg R Reichborn-Kjennerud T Jacobsen BK Sex differences in mortality among patients admitted with affective disorders in North Norway: a 33-year prospective register study. Bipolar Disord (2016) 18(3):272–81. 10.1111/bdi.12389 27226265

[23] Ösby U Brandt L Correia N Ekbom A Sparén P Excess mortality in bipolar and unipolar disorder in Sweden. Arch Gen Psychiatry (2001) 58(9):844–50. 10.1001/archpsyc.58.9.844 11545667

[24] Tidemalm D Långström N Lichtenstein P Runeson B Risk of suicide after suicide attempt according to coexisting psychiatric disorder: Swedish cohort study with long term follow-up. BMJ (2008) 337:a2205. 10.1136/bmj.a2205 19018040 PMC2590902

[25] Haglund A Lysell H Larsson H Lichtenstein P Runeson B Suicide Immediately After Discharge From Psychiatric Inpatient Care: A Cohort Study of Nearly 2.9 Million Discharges. J Clin Psychiatry (2019) 80(2):pii: 18m12172. 10.4088/JCP.18m12172 30758922

[26] Isometsä E Sund R Pirkola S Post-discharge suicides of inpatients with bipolar disorder in Finland. Bipolar Disord (2014) 16(8):867–74. 10.1111/bdi.12237 25056223

[27] Toffol E Hätönen T Tanskanen A Lönnqvist J Wahlbeck K Joffe G Lithium is associated with decrease in all-cause and suicide mortality in high-risk bipolar patients: A nationwide registry-based prospective cohort study. J Affect Disord (2015) 183:159–65. 10.1016/j.jad.2015.04.055 26005778

[28] Hansson C Joas E Pålsson E Hawton K Runeson B Landén M Risk factors for suicide in bipolar disorder: a cohort study of 12 850 patients. Acta Psychiatr Scand (2018) 138(5):456–63. 10.1111/acps.12946 PMC6220973 30076611

[29] Høyer EH Licht RW Mortensen PB Risk factors of suicide in inpatients and recently discharged patients with affective disorders. A case-control study. Eur Psychiatry (2009) 24(5):317–21. 10.1016/j.eurpsy.2008.03.011 19410433

[30] Aaltonen KI Isometsä E Sund R Pirkola S Risk factors for suicide in depression in Finland: first-hospitalized patients followed up to 24 years. Acta Psychiatr Scand (2019) 139(2):154–63. 10.1111/acps.12990 30480317

[31] Chung DT Ryan CJ Hadzi-Pavlovic D Singh SP Stanton C Large MM Suicide Rates After Discharge From Psychiatric Facilities: A Systematic Review and Meta-analysis. JAMA Psychiatry (2017) 74(7):694–702. 10.1001/jamapsychiatry.2017.1044 28564699 PMC5710249

[32] Chung D Hadzi-Pavlovic D Wang M Swaraj S Olfson M Large M Meta-analysis of suicide rates in the first week and the first month after psychiatric hospitalisation. BMJ Open (2019) 9(3):e023883. 10.1136/bmjopen-2018-023883 PMC6475206 30904843

[33] Runeson B Tidemalm D Dahlin M Lichtenstein P Långström N Method of attempted suicide as predictor of subsequent successful suicide: national long term cohort study. BMJ (2010) 341:c3222. 10.1136/bmj.c3222 20627975 PMC2903664

[34] Bergen H Hawton K Waters K Ness J Cooper J Steeg S How do methods of non-fatal self-harm relate to eventual suicide? J Affect Disord (2012) 136(3):526–33. 10.1016/j.jad.2011.10.036 22127391

[35] Olfson M Wall M Wang S Crystal S Gerhard T Blanco C Suicide Following Deliberate Self-Harm. Am J Psychiatry (2017) 174(8):765–74. 10.1176/appi.ajp.2017.16111288 28320225

[36] Isometsä E Psychological autopsy studies - a review. Eur Psychiatry (2001) 16:379–85. 10.1016/S0924-9338(01)00594-6 11728849

[37] Holma KM Melartin TK Haukka J Holma IA Sokero TP Isometsä ET Incidence and predictors of suicide attempts in DSM-IV major depressive disorder: a five-year prospective study. Am J Psychiatry (2010) 167:801–8. 10.1176/appi.ajp.2010.09050627 20478879

[38] Holma M Haukka J Suominen K Valtonen HM Mantere O Melartin TK Differences in incidence of suicide attempts between bipolar I and II disorders and major depressive disorder. Bipolar Disord (2014) 16:652–61. 10.1111/bdi.12195 24636453

[39] Pallaskorpi S Suominen K Ketokivi M Valtonen H Arvilommi P Mantere O Incidence and predictors of suicide attempts in bipolar I and II disorders: A 5-year follow-up study. Bipolar Disord (2017) 19:13–22. 10.1111/bdi.12464 28176421

[40] Nelson JC Bickford D Delucchi K Fiedorowicz JG Coryell WH Risk of Psychosis in Recurrent Episodes of Psychotic and Nonpsychotic Major Depressive Disorder: A Systematic Review and Meta-Analysis. Am J Psychiatry (2018) 175(9):897–904. 10.1176/appi.ajp.2018.17101138 29792050

[41] Holma KM Holma IA Melartin TK Rytsälä HJ Isometsä ET Long-term outcome of major depressive disorder in psychiatric patients is variable. J Clin Psychiatry (2008) 69:196–205. 10.4088/JCP.v69n0205 18251627

[42] Walby FA Myhre MØ Kildahl AT Contact With Mental Health Services Prior to Suicide: A Systematic Review and Meta-Analysis. Psychiatr Serv (2018) 69(7):751–9. 10.1176/appi.ps.201700475 29656710

[43] Agerbo E High income, employment, postgraduate education, and marriage: a suicidal cocktail among psychiatric patients. Arch Gen Psychiatry (2007) 64(12):1377–84. 10.1001/archpsyc.64.12.1377 18056545

[44] Melartin TK Rytsälä HJ Leskelä US Lestelä-Mielonen PS Sokero TP Isometsä ET Current comorbidity of psychiatric disorders among DSM-IV major depressive disorder patients in psychiatric care in the Vantaa Depression Study. J Clin Psychiatry (2002) 63:126–34. 10.4088/JCP.v63n0207 11874213

[45] Mantere O Suominen K Melartin T Valtonen H Arvilommi P Leppämäki S Differences in patterns of axis I and II comorbidity of bipolar I vs. II vs. major depressive disorders. J Clin Psychiatry (2006) 67:584–93. 10.4088/JCP.v67n0409 16669723

[46] Simon GE Johnson E Lawrence JM Rossom RC Ahmedani B Lynch FL Predicting Suicide Attempts and Suicide Deaths Following Outpatient Visits Using Electronic Health Records. Am J Psychiatry (2018) 175(10):951–60. 10.1176/appi.ajp.2018.17101167 PMC6167136 29792051

[47] Simon GE Yarborough BJ Rossom RC Lawrence JM Lynch FL Waitzfelder BE Self-Reported Suicidal Ideation as a Predictor of Suicidal Behavior Among Outpatients With Diagnoses of Psychotic Disorders. Psychiatr Serv (2019) 70(3):176–83. 10.1176/appi.ps.201800381 PMC6520048 30526341

[48] Gong J Simon GE Liu S Machine learning discovery of longitudinal patterns of depression and suicidal ideation. PLoS One (2019) 14(9):e0222665. 10.1371/journal.pone.0222665. 31539408 PMC6754154

[49] Belsher BE Smolenski DJ Pruitt LD Bush NE Beech EH Workman DE Prediction Models for Suicide Attempts and Deaths: A Systematic Review and Simulation. JAMA Psychiatry (2019) 76(6):642–51. 10.1001/jamapsychiatry.2019.0174 30865249

